# Effectiveness of Immune Checkpoint Inhibitor with Anti-PD-1 Monotherapy or in Combination with Ipilimumab in Younger versus Older Adults with Advanced Melanoma

**DOI:** 10.3390/curroncol30100646

**Published:** 2023-09-30

**Authors:** Taylor E. Woo, Igor Stukalin, Philip Q. Ding, Siddhartha Goutam, Michael Sander, Benjamin Ewanchuk, Winson Y. Cheung, Daniel Y. C. Heng, Tina Cheng

**Affiliations:** 1 Department of Medicine, Division of Dermatology, University of Calgary, Calgary, AB T2N 1N4, Canada; 2 Department of Oncology, Division of Medical Oncology, Tom Baker Cancer Centre, Calgary, AB T2N 4N2, Canada; istukali@ucalgary.ca (I.S.); philip.ding@albertahealthservices.ca (P.Q.D.); goutam@ualberta.ca (S.G.); dr.michael.s.sander@gmail.com (M.S.); ben.ewanchuk@ucalgary.ca (B.E.); winson.cheung@ucalgary.ca (W.Y.C.); daniel.heng@albertahealthservices.ca (D.Y.C.H.)

**Keywords:** melanoma, immune checkpoint inhibitor, nivolumab, pembrolizumab, ipilimumab plus nivolumab, age, older adults, survival

## Abstract

**Background**: The majority of melanoma is diagnosed in individuals between 55 and 84 years old. Current data varied in reporting differences in survival outcomes amongst different age groups. **Methods:** A retrospective, multi-center, provincial cohort database was used to investigate the relationship between age (<65 or ≥65 years old) and overall survival. Patients must have had histologically confirmed locally advanced or metastatic melanoma and had to have received at least one cycle of immunotherapy (single agent nivolumab, pembrolizumab, or combination ipilimumab plus nivolumab). **Results:** From August 2013 to May 2020, we identified 497 patients (median age = 64 [range 12–96 years]; 65.2% men; 36.4% with a *BRAF* mutation (V600E and V600K)). Of these, 260 were < 65 years old, and 237 were ≥65 years old. A total of 39.1% of the patients in the younger cohort received combination ICI compared with 10.2% in the older cohort, and the difference was statistically significant. Median survival amongst individuals aged ≥65 years old was shorter compared to individuals <65 years old, with a median overall survival of 17.1 (95% CI 12.3–22.9 months) months and 22.2 months (95% CI 18.7–33.8 months), respectively (*p* = 0.04), at a median follow-up of 34.4 months (range: 1.84–81.4 months). The survival difference was present in the cutaneous melanoma cohort where median OS was 18.2 months (95% CI 12.3–30.4 months) in patients ≥65 years old and 23.8 months (95% CI 19.2–48.2 months) in patients <65 years old, *p* = 0.04. There were no significant differences by age in the non-cutaneous melanoma cohort. A combination of nivolumab plus ipilimumab was associated with an improved overall survival hazard ratio of 0.48 (95% CI 0.36–0.65) as compared to anti-PD-1 monotherapy alone (*p* < 0.001). In the cutaneous cohort treated with anti-PD-1 monotherapy (*n* = 306), no significant differences were seen with median OS at 16.1 months (95% CI 11.4–25.7 months) in patients ≥65 years old and 17.1 months (95% CI 12.0–22.2 months) in patients <65 years old (*p* = 0.84). Tumor response to anti-PD-1 was higher in the older patients compared with the response in younger patients with cutaneous melanoma. **Conclusions:** Older melanoma patients have similar survival compared with younger patients after receiving the same treatment with anti-PD-1 monotherapy. The superior survival observed in the younger patients is possibly related to the higher utilization of combination ICI. Tumor response to immunotherapy is superior in older patients with cutaneous melanoma; however, younger patients may improve their survival by using combination ICI.

## 1. Introduction

Melanoma is a disease of global significance, accounting for 1.7% of cancer diagnoses, and its incidence is projected to increase worldwide [1,2,3]. A study by Saginala et al. observed that the incidence of melanoma in the US increased by 320% between 1975 and 2018 [3]. The development of immune checkpoint inhibitors (ICI) has revolutionized the treatment paradigm for melanoma and has led to significant improvement in overall survival (OS) and progression-free survival (PFS) [4,5,6]. The update of CheckMate 067 clinical trial for advanced melanoma at 6.5-year follow-up reported an OS of 72.1 months in patients treated with combination anti-programmed cell death protein 1 (anti-PD-1) nivolumab with cytotoxic T-cell lymphocyte-antigen 4 (CTLA4) ipilimumab and 36.9 months in patients treated with single-agent nivolumab [5].

The majority of melanoma is diagnosed in individuals between 55 and 84 years old [3], and age 65 years or older is often used to define melanoma in older adults [7,8,9]. Melanoma in older adults is underrepresented in clinical trials due to multiple factors, including concerns for toxicity tolerance [7]. There are fundamental differences in the tumor biology and host immune function in melanoma developed at different ages [10]. Melanoma in older adults is different in molecular pathogenesis. Age-related decline of the immune system and chronic pro-inflammatory aging may modify the efficacy and toxicity of ICI [8]. Age may also be a surrogate marker for comorbidities and declining global function. Current data varied in reporting differences in survival outcomes amongst different age groups, with some studies reporting comparable efficacy of ICI between older and younger adults with solid tumors, including melanoma [7,11,12,13], while others reported better outcomes in older adults [14]. We conducted this retrospective multi-center cohort study to understand the potential effects of age on treatment outcomes in advanced melanoma after receiving ICI.

## 2. Materials and Methods

### 2.1. Study Population and Design

The Alberta Immunotherapy Database (AID) is a database of patients who have received systemic therapies for melanoma in the Province of Alberta (two tertiary cancer centers (Tom Baker Cancer Centre and Cross Cancer Institute) and four regional cancer centers (Central Alberta Cancer Center; Grande Prairie Cancer Center; Jack Ady Cancer Center; and Margery E. Yuill Cancer Center)), Canada. The data were obtained through registry, pharmacy, and/or consecutive clinic lists by individual retrospective chart reviews using standardized database templates.

Patients who had histologically confirmed unresectable or metastatic melanoma and received at least one cycle of ICI (single agent nivolumab, pembrolizumab, or combination ipilimumab and nivolumab) between August 2013 and May 2020 were included in this study. We collected disease characteristics, clinical and biochemical parameters at baseline of treatment, date of first treatment, best radiographic response, length of treatment, adverse events, and date of death or last follow-up. Cutaneous melanoma included melanoma arising from the skin, including acral melanoma and melanoma of unknown primary. Non-cutaneous melanoma included patients with a diagnosis of uveal and mucosal melanoma. Clinical staging was based on the criteria of the American Joint Committee on Cancer (AJCC) 8th edition [15]. Statistical analysis was conducted in February 2022. Institutional review board ethics approval was obtained prior to undertaking data collection (HREBA.CC-19-0380).

### 2.2. Outcomes of Interest

The primary endpoint of this study was OS, defined as the time from commencement of ICI to death from any cause or censor at the time of last follow-up. The cutoff point for data collection was 31 December 2021. The Older cohort was defined as age ≥65 years and the younger cohort was defined as age <65 years based on the prior literature [7,8,9]. We examined OS specifically in patients <65 and ≥65 years of age. Additional outcomes included treatment duration (TD), which was calculated from the start of ICI initiation until treatment cessation or censored at the time of the last follow-up. Objective response rate (ORR) was defined as the proportion of patients achieving a complete or partial response radiographically based on the Response Evaluation Criteria in Solid Tumors (RECIST) version 1.1 [6].

### 2.3. Statistical Analysis

We used descriptive statistics to summarize demographic and clinical characteristics of the overall cohort and patient subgroups, with categorical variables presented as frequencies (percentages) and continuous variables expressed as median (interquartile range). We performed subgroup analyses after stratifying patients by age at treatment initiation, <65 and ≥65 years old. We compared the characteristics between patient subgroups using Pearson’s chi-squared test for categorical variables with all cell sizes ≥5 and Fisher’s exact test for categorical variables with any cell size <5.

Using the Kaplan–Meier method, we estimated median OS and TD for each patient subgroup and plotted corresponding Kaplan–Meier curves. In addition to the overall cohort, we conducted survival and time-to-treatment discontinuation analysis with focus on each of the two major melanoma types, cutaneous and non-cutaneous. We assessed differences between patient subgroups using the log-rank test.

We built Cox proportional hazard models to identify characteristics associated with OS for the overall cohort as well as among age and melanoma-type subgroups. We used univariate Cox regression to assess the individual predictive value of each factor. We subsequently included in multivariable models the candidate predictors that showed statistical significance in univariate analysis and those with high clinical relevance.

To further assess differences in treatment effectiveness within each age group, we use created propensity score-matched cohorts with the previously identified candidate predictors as covariates. For combination ICI vs. anti-PD-1 monotherapy patients, we performed 1:1 nearest neighbor matching without replacement with caliper width of 0.2 standard deviations of the logit of the propensity score. Balance was assessed in the matched cohorts using standardized mean differences with thresholds of <0.10 and <0.20 and comparison with the pre-matching cohorts.

All statistical tests were two-sided, and the significance level was defined a priori as <0.05. All analyses were performed using R [6].

## 3. Results

### 3.1. Patient Demographics

Of the 497 patients who received an anti-PD-1 alone or in combination with ipilimumab, 324 (65.2%) were male, and the median age at treatment initiation was 64 years (range: 12–96 years) (Table 1). Among them, 418 (84.1%) patients had cutaneous melanoma, and 79 (15.9%) had non-cutaneous melanoma. A total of 151 (36.4%) patients harbored a *BRAF* mutation (V600E, V600K). A total of 429 (86.5%) patients had an ECOG performance status of 0 or 1.

There are no significant differences between patient cohort age <65 years (*n* = 260) and cohort age ≥65 years (*n* = 237) in gender, ECOG performance status, melanoma type, tumor burden and metastatic involvement, lactate dehydrogenase (LDH), and derived neutrophil to lymphocyte ratio (dNLR) (Table 1). *BRAF* mutation was more frequent in patients aged <65 years (43.8%) compared to those aged ≥65 years (28.1%), *p* = 0.001. Amongst the entire cohort, 29.4% of the patients had received prior treatments (mostly BRAF-targeted therapy); 34.6% of patients <65 years of age and 23.6% of patients ≥65 years of age had received prior treatments. The median time from diagnosis of metastatic disease to the initiation of immunotherapy was 4 weeks (interquartile range 3–10 weeks) in patients <65 years and 5 weeks (interquartile range 3–10 weeks) in patients ≥65 years of age (*p* = 0.32).

### 3.2. Treatments

Overall, 368 (74.8%) patients received an anti-PD-1 monotherapy, and 124 (25.2%) patients received a combination of ipilimumab plus nivolumab. Anti-PD-1 monotherapy was used more frequently in patients ≥ 65 years (*n* = 212, 89.8%) compared with patients <65 years (*n* = 156, 60.9%), *p* < 0.001. Among patients who received PD-1 monotherapy, *BRAF*+ disease was more common in adults aged ≥65 as compared to those <65, with 39% of patients harboring the *BRAF* mutation (*p* = 0.01). Ipilimumab plus nivolumab were used more frequently in patients <65 years (*n* = 100, 39.1%) compared with patients ≥65 years (*n* = 24, 10.2%), *p* < 0.001. More patients (*n* = 181, 76.4%) in patients ≥ 65 years received ICI first line compared to the 170 (65.4%) in patients <65 years, *p* < 0.007.

In the entire cohort (*n* = 497), median TD was longer at 4.4 months (95% CI 3.7–5.6 months) in patients <65 years of age compared with 3.5 months (95% CI 2.8–5 months) in patients ≥65 years old, *p* = 0.0053. Differences in treatment duration were observed in the cutaneous melanoma cohort (with median TD at 4.4 months (95% CI 3.2–6 months) in patients <65 years and at 4.1 months (95% CI 2.9–5.1 months) in patients ≥65 years old (*p* = 0.016). There was no difference in TD in the non-cutaneous cohort. When patients on combination ICI therapy were excluded (*n* = 368), no significant differences in treatment duration were observed between individuals aged <65 years and ≥65 years at 4.8 months (95% CI 3.7–6.2 months) versus 4.3 months (95% CI 3–5.1 months), respectively, *p* = 0.07 (Supplemental Figure S1).

### 3.3. Survival

With a median follow-up of 34.43 months (range: 1.84–81.35 months), 190 (38.4%) patients were alive, and the median OS was 19.8 months (range, 17.1–23.8).

**Figure 1:**
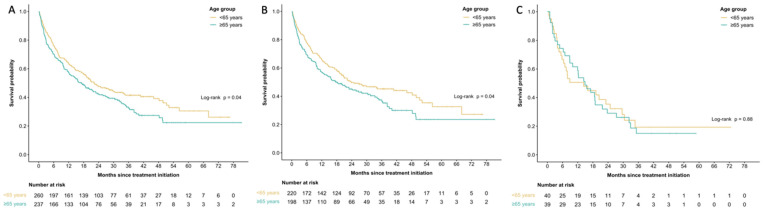
Kaplan–Meier curves for median overall survival amongst patients aged <65 and ≥65 years old for all melanoma patients (**A**), patients with cutaneous melanoma only (**B**), and non-cutaneous melanoma only (**C**).

In the entire cohort (*n* = 497), survival was shorter in the older cohort with median OS at 17.1 months (95% CI 12.3–22.9 months) in patients ≥65 years old and 22.2 months (95% CI 18.7–33.8 months) in patients <65 years, *p* = 0.04 (Figure 1A). The survival difference was driven by the cutaneous melanoma cohort where median OS was 18.2 months (95% CI 12.3–30.4 months) in patients ≥65 years old and 23.8 months (95% CI 19.2–48.2 months in patients <65 years old, *p* = 0.04 (Figure 1B). No significant differences were seen in the non-cutaneous melanoma cohort, with median OS at 14.9 months (95% CI 10.0–23.7 months) in patients ≥65 years old and 14.4 months (95% CI 6.9–29.6 months) in patients < 65 years old (*p* = 0.88) (Figure 1C).

In the cutaneous cohort treated with anti-PD-1 monotherapy (*n* = 306), no significant differences were seen in survival with median OS at 16.1 months (95% CI 11.4–25.7 months) in patients ≥65 years old and 17.1 months (95% CI 12.0–22.2 months) in patients <65 years old (*p* = 0.84) (Supplemental Figure S2A). In the non-cutaneous cohort treated with anti-PD-1 monotherapy (*n* = 62), no significant differences were seen in survival with median OS at 8.0 months (95% CI 5.4–24.8 months) in patients <65 years old and 14.9 months (95% CI 8.9–23.7 months) in patients ≥65 years old (*p* = 0.57) (Supplemental Figure S2B).

### 3.4. Efficacy

Objective response was observed in 200 patients (40.2%) of the entire cohort of 497 patients, with 60 patients (15.5%) achieving complete response. ORR was 45% (CR17.4%) in the cutaneous cohort and 15.2% (CR 6.2%) in the non-cutaneous cohort. In the cutaneous cohort treated with anti-PD-1 alone or in combination with ipilimumab (*n* = 418), ORR was 47% in patients ≥65 years old and 43.2% in patients <65 years old, *p* = 0.44 (of note, there were more patients treated with combination ICI in the younger cohort). In the cutaneous cohort treated with anti-PD-1 monotherapy (*n* = 306), ORR was higher in age ≥65 years at 47.5% versus 36.4% in age <65 years, *p* = 0.05 with the best response achieved favoring the older cohort, *p* = 0.008. Response rate and best response were not different between the two age cohorts in non-cutaneous patients.

Fifty-nine patients with *BRAF*-mutated melanoma received subsequent therapy after failing ICI. BRAF-targeted therapy was used most at 84.8% (81.4% in patients <65 years and 93.8% in patients 65 years), followed by pembrolizumab at 6.8%, ipilimumab at 3.4%, and clinical trial at 1.7%. The combination of ipilimumab plus nivolumab was only used in one patient who was in the younger cohort. In patients <65 years old, nine patients (20.9%) received a second subsequent therapy, and one (2.3%) patient received a third therapy. No individuals ≥65 years old received a second or third therapy.

### 3.5. Analysis of Clinical Factors Associated with Survival

We examined factors associated with differential survival observed between the younger and older cohorts in the overall cohort (Supplemental Table S1) and the cutaneous cohort (Supplemental Table S2). As the superior survival in the age <65 years cohort was observed in the cutaneous cohort while the non-cutaneous cohort revealed no survival difference, we performed univariate and multivariate analyses of baseline clinical factors associated with OS in the cutaneous cohort (Supplemental Table S1).

Age < 65 years, normal LDH, dNLR < 3, ECOG performance status < 2, stage M1a/1b, combination ipilimumab plus nivolumab, and first-line immunotherapy were significantly associated with improved OS in univariate analysis (Supplemental Table S2). However, age was not independently associated with OS (*p* = 0.59), and the combination of ipilimumab plus nivolumab showed a trend of independent association (*p* = 0.07) in multivariate analysis. Normal LDH, dNLR ≤3, ECOG performance status <2, stage M1a/1b, and first-line IO remained independently associated with OS (Supplemental Table S2).

We then performed univariate and multivariate analyses stratifying the cutaneous cohort by age <65 years and ≥65 years old (Table 2). Normal LDH and first-line immunotherapy were independently associated with better OS in multivariate analysis. dNLR ≤ 3 was independently associated with better survival in cohort age <65 years, and ECOG performance status was independently associated with better survival in cohort age ≥65 years. Compared to anti-PD-1 monotherapy, combination ICI was associated with superior survival in age <65 years in both univariate (*p* < 0.001) and multivariate analyses (*p* = 0.01). Combination ICI was not associated with improved survival in the age ≥65 years group in univariate (*p* = 0.25) (Supplemental Figure S3) and multivariate (*p* = 0.41) analyses.

**Figure 2:**
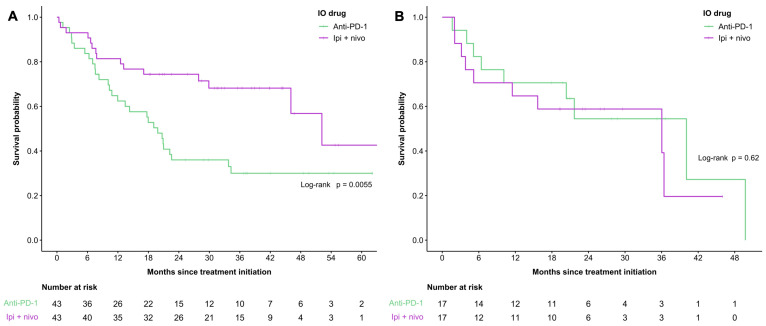
Overall survival of propensity score-matched cohort by treatment group (anti-PD-1 only versus combination therapy) in patients aged <65 years with cutaneous melanoma (**A**) and patients aged ≥65 years with cutaneous melanoma (**B**).

Propensity score matching was completed for patients with cutaneous melanoma stratified by age group using sex, ECOG performance score, LDH, dNLR, M stage, and treatment line as covariates (Figure 2, Supplemental Tables S3 and S4). After matching, there were 86 patients in the <65 years cohort (*n* = 43 in each treatment group) (Figure 2A) and 34 patients in the ≥65 years old cohort (*n* = 17 in each treatment group) (Figure 2B). Overall survival amongst patients <65 years was 19.8 months and 52.2 months in the anti-PD-1 and combination therapy groups, respectively (*p* = 0.0055). No significant differences were found amongst individuals aged ≥65 years of age amongst the propensity score-matched cohort, with an OS of 40.1 months in the anti-PD-1 cohort and 36 months in the combination therapy subgroup (*p* = 0.62). Propensity score matching yielded sizeable improvements in the balance of important baseline characteristics between treatment groups, with 33% of covariates having imbalances at the <0.20 SMD threshold in the <65 years cohort and all covariates being balanced at the <0.10 threshold in the ≥65 years cohort.

## 4. Discussion

In this multi-center retrospective study, we investigated the association of age with survival in patients who received ICI for advanced melanoma. We report superior survival in patients under the age of 65 years in comparison to patients 65 years or older in the entire cohort and in the cutaneous melanoma cohort. Age as a factor is not independently associated with survival. Factors that are independently associated with better survival are the use of ICI at the first line, normal serum LDH, a favorable dNLR (≤3), a better ECOG performance status (<2), and an earlier metastatic stage (M1a/1b). Combination ICI is independently associated with improved survival in the younger cohort, but it is not associated with improved survival in the older cohort in univariate and multivariate analyses.

Our study suggests that the superior survival observed in the younger patients is secondary to the higher utilization of combination ICI. A total of 39.1% of the patients in the younger cohort received combination ICI compared with 10.2% in the older cohort, and the difference was statistically significant. There were no statistically significant differences among the two age cohorts in other important factors at baseline, including serum LDH, dNLR, ECOG performance status, and metastatic stage. There was a statistically significant difference in the line of treatment between the two age cohorts, but the difference favored the older cohort, with more older patients receiving first-line ICI. First-line ICI, in comparison to frontline targeted therapy, is associated with longer survival in multiple studies. Excluding patients treated with combination ICI, there was no survival difference between the younger and older cohorts.

It is worth noting that the older melanoma population did not have inferior survival compared with the younger cohort when treatment was restricted to anti-PD-1 monotherapy (excluding combination ICI). This is an important observation, as older age has been consistently associated with poorer outcomes in the early stages of melanoma in multiple prior studies [16,17,18,19,20]. We further observed superior tumor response to anti-PD-1 monotherapy in the older cutaneous patients compared with response in younger patients.

This improved response may be secondary to age-related differences in melanoma tumor biology and host immune function in response to anti-PD-1. Melanoma is an immunogenic cancer due to UV-induced high tumor mutation burden, resulting in neoantigens that can be recognized as foreign by the immune system [21]. Older individuals commonly develop high chronically sun-damaged (CSD) melanoma arising in the chronically sun-exposed skin, while younger individuals develop low-CSD melanoma primarily arising in the intermittently sun-exposed skin [10]. High-CSD melanomas have a threefold higher tumor mutation burden than low-CSD melanomas [21], and a higher mutation burden is associated with enhanced sensitivity to ICIs, including anti-PD-1 [22]. Independent of a more complex mutational landscape in older patients, the aged tumor microenvironment was found to contribute to the likelihood of response to anti-PD-1, with the odds of progressing decreased by 13% for each decade of age at ICI initiation [14]. Age-related decline in the immune system (immunosenescence) was found to result in an increase in immune checkpoint protein and, therefore, a greater response to anti-PD-1 [23]. Among melanoma subtypes, higher PD-L1 expression was found in cumulative sun damage-related melanomas in older patients [24]. Clinically, Ben-Betzalel et al. found response rates to anti-PD-1 exceeding 62% in patients 65 years or older and 73.9% in patients 80 years or older with metastatic melanoma [12]. Other studies reported no age-related differences in outcomes; some possible reasons for the observation are a small sample size [25], the inclusion of multiple solid tumors [7,13], or primarily the inclusion of anti-CTLA4 treated patients [11].

Although the younger patients may have less immunotherapy-sensitive melanoma, this can be overcome by combination ICI. In the CheckMate 067 phase III study comparing combination ipilimumab plus nivolumab versus nivolumab or ipilimumab monotherapy, the benefit of combination ICI over anti-PD-1 was observed in the age <65 years group and not in the age ≥65 years group [26]. In our study, combination ICI is independently associated with improved survival in the younger cohort, whereas it is not associated with improved survival in the older cohort in univariate and multivariate analyses.

The association between dNLR < 3, normal LDH, ECOG < 2, and M1a/1b stage with improved survival outcomes is in keeping with previous studies, which have identified that an elevated LDH and higher dNLR to be associated with worse OS [27,28]. Both an elevated LDH and elevated dNLR are associated with a more extensive tumor burden and a heightened pro-inflammatory state, thereby leading to worse survival outcomes. In addition, elevated ECOG is an established prognostic factor linked to decreased OS in several malignancies, including non-small lung cancer, melanoma, and renal cell carcinoma patients treated with immunotherapy [29,30]. Lastly, patients with M1c/d disease, particularly those with involvement of the liver and brain, tend to have worse responses to ICI and are also associated with worse outcomes [31,32,33].

Our findings, combined with the current literature, may provide useful information to physicians. Age is not an independent predictor of benefit to ICI in advanced cutaneous melanoma. Furthermore, chronological age is a continuous variable and an imprecise measurement of the host’s biological condition. However, there are some general considerations. Anti-PD-1 monotherapy may allow the more senior older patients to achieve excellent survival without the risk of added toxicity from combination ICI as their melanoma is more responsive to ICI, and their tolerance of toxicity may be lower. The CheckMate 067 study, on prolonged follow-up, revealed similar survival should a response be achieved regardless of treatment with either anti-PD-1 alone or combination ICI. On the other hand, melanoma in younger adults, on relative terms, is less immune-responsive, and combination ICI may be the preferred option for most patients. In addition, patients with BRAF V600-mutated melanoma, elevated LDH, and brain metastases have been shown to draw a higher magnitude of benefit in survival from combination ICI in comparison to anti-PD-1 monotherapy based on subgroup analyses of the CheckMate 067 study [5,34]. Other considerations in making individualized decisions include clinical factors such as ECOG PS, extent of disease, presence of hepatic metastasis, elevated or normal risk for developing and tolerance of irAE, and patient preference.

Although older patients are more likely to die from reasons other than melanoma, a prior study of 254 melanoma patients treated with anti-PD-1 (92.5%) or anti-PD-L1 (7.5%) found similar survival (OS; PFS) regardless of the patient’s age. Comorbidity measured by the Charlson Comorbidity Index was not associated with OS and PFS [25]. A study by Perier-Muzet et al. found that melanoma patients older than 65 years, despite having higher cumulative illness rating scores, had better PFS and OS after receiving ICI, particularly after receiving an anti-PD-1 treatment [35]. A large study of 562 melanoma patients treated with pembrolizumab reported significantly higher PFS and OS in patients between 70 and 80 years compared with younger patients, but the age effect disappeared after age above 80 years, probably due to an increase in significant comorbidity [36]. Future studies will be needed to look at disease-specific survival.

Interpretation of our data in non-cutaneous melanoma is limited due to the small sample size. Non-cutaneous melanoma carries a comparatively worse prognosis and variable immunotherapy response, which is consistent with prior reports. Our data suggest combination ICI is significantly superior to anti-PD-1 monotherapy in this population.

Additional limitations of our study include the retrospective design of our analysis and the uneven distribution of baseline characteristics in the different age groups. Particularly, missing clinical information in charting is a limitation. In addition, we selected 65 years old as our cutoff and treated this as a dichotomous variable, as performed by other studies [7,8,9]. Although age is a continuous variable, dichotomizing it for the purpose of survival analyses is common practice [9,25]. Only 24 patients (10.2%) in the older cohort received combination ICI, and this may affect data interpretation in this cohort.

## 5. Conclusions

Melanoma diagnoses among the elderly are expected to increase [3]. Understanding the effect of age on melanoma treatment outcomes for ICI is immensely important and may be used in addition to other important disease and host factors to decide on the most appropriate treatment for an individual. Older melanoma patients may have superior tumor response to immunotherapy, while young patients may benefit from more aggressive ICI. The effect of age on clinical outcomes may vary among different tumors and needs to be examined individually.

## Figures and Tables

**Table 1:** Patient demographics and clinical and disease characteristics at baseline.

Characteristic	*N*	Overall, *N* = 497	Age at Treatment Initiation, Years		*p*-Value
			<65, *N* = 260	≥65, *N* = 237	
**Sex**	**497**				0.11
Female		173 (34.8%)	99 (38.1%)	74 (31.2%)	
Male		324 (65.2%)	161 (61.9%)	163 (68.8%)	
**ECOG status**	496				0.11
<2		429 (86.5%)	231 (88.8%)	198 (83.9%)	
≥2		67 (13.5%)	29 (11.2%)	38 (16.1%)	
**Melanoma type**	497				0.74
Cutaneous		418 (84.1%)	220 (84.6%)	198 (83.5%)	
Non-cutaneous		79 (15.9%)	40 (15.4%)	39 (16.5%)	
**BRAF mutation**	415				0.001
No		264 (63.6%)	123 (56.2%)	141 (71.9%)	
Yes		151 (36.4%)	96 (43.8%)	55 (28.1%)	
**LDH**	404				0.99
Normal		273 (67.6%)	144 (67.6%)	129 (67.5%)	
Elevated >ULN		131 (32.4%)	69 (32.4%)	62 (32.5%)	
**dNLR**	477				0.09
≤3		385 (80.7%)	210 (83.7%)	175 (77.4%)	
>3		92 (19.3%)	41 (16.3%)	51 (22.6%)	
**Number of organ sites with metastasis**	497				0.98
<3		331 (66.6%)	173 (66.5%)	158 (66.7%)	
≥3		166 (33.4%)	87 (33.5%)	79 (33.3%)	
**M stage**	497				0.71
1a/1b		241 (48.5%)	124 (47.7%)	117 (49.4%)	
1c/1d		256 (51.5%)	136 (52.3%)	120 (50.6%)	
**Autoimmune condition**	496				0.13
No		438 (88.3%)	235 (90.4%)	203 (86.0%)	
Yes		58 (11.7%)	25 (9.6%)	33 (14.0%)	
**IO drug**	492				<0.001
Anti-PD-1 *		368 (74.8%)	156 (60.9%)	212 (89.8%)	
Ipilimumab + nivolumab		124 (25.2%)	100 (39.1%)	24 (10.2%)	
**Treatment line**	497				0.007
First		351 (70.6%)	170 (65.4%)	181 (76.4%)	
Other		146 (29.4%)	90 (34.6%)	56 (23.6%)	

* = Pembrolizumab or Nivolumab.

**Table 2:** Univariate and multivariate analyses of baseline factors associated with overall survival in cutaneous patients stratified by age.

Characteristic	Univariate Cox Regression Model				Multivariate Cox Regression Model			
	Age < 65 Years		Age ≥ 65 Years		Age < 65 Years		Age ≥ 65 Years	
	OS HR (95% CI)	*p*-Value	OS HR (95% CI)	*p*-Value	OS HR (95% CI)	*p*-Value	OS HR (95% CI)	*p*-Value
**Sex**								
Female (reference) vs. Male	1.18 (0.82–1.70)	0.38	0.96 (0.64–1.42)	0.83	1.36 (0.90–2.07)	0.15	0.93 (0.59–1.48)	0.77
**dNLR**								
≤3 (reference) vs. >3	2.50 (1.61–3.87)	**<0.001**	1.80 (1.20–2.70)	**0.005**	1.88 (1.12–3.18)	**0.02**	1.35 (0.83–2.18)	0.23
**LDH**								
Normal (reference) vs. Elevated	2.22 (1.49–3.30)	**<0.001**	2.38 (1.59–3.55)	**<0.001**	2.00 (1.31–3.06)	**0.001**	2.09 (1.37–3.18)	**<0.001**
**ECOG status**								
<2 (reference) vs. ≥2	2.30 (1.39–3.80)	**0.001**	3.05 (1.95–4.75)	**<0.001**	1.30 (0.69–2.44)	0.42	2.86 (1.61–5.08)	**<0.001**
**M stage**								
1a/1b (reference) vs. 1c/1d	2.31 (1.61–3.32)	**<0.001**	1.51 (1.06–2.15)	**0.02**	1.53 (1.00–2.36)	0.05	1.24 (0.80–1.92)	0.34
**BRAF mutation**								
No (reference) vs. Yes	1.35 (0.93–1.96)	0.12	1.10 (0.73–1.65)	0.66				
**IO drug**								
Anti-PD-1 (reference) vs. Ipilimumab + nivolumab	0.47 (0.32–0.71)	**<0.001**	0.68 (0.36–1.31)	0.25	0.51 (0.30–0.87)	**0.01**	1.35 (0.66–2.75)	0.41
**Treatment line**								
First (reference) vs. Other	2.49 (1.74–3.56)	**<0.001**	**1.68 (1.15–2.45)**	**0.007**	1.73 (1.08–2.78)	**0.02**	1.84 (1.14–2.99)	**0.01**

## Data Availability

The data presented in this study are available on request from the corresponding author.

## Supplementary Materials

The following supporting information can be downloaded at https://www.mdpi.com/article/10.3390/curroncol30100646/s1. Figure S1: Kaplan–Meier curves of treatment duration amongst patients aged <65 and ≥65 years old, excluding combination immunotherapy for all melanoma (A), cutaneous melanoma only (B), and non-cutaneous melanoma only (C). Figure S2: Kaplan–Meier curves for median overall survival amongst patients aged <65 and ≥65 years old, when patients receiving combination immunotherapy were excluded for cutaneous melanoma patients only (A) and non-cutaneous melanoma only (B). Figure S3: Kaplan–Meier curves for median overall survival amongst patients aged <65 and ≥65 years old when stratified by patients treated with combination immunotherapy (ipilimumab and nivolumab). Table S1: Univariate and multivariate analyses of baseline factors associated with overall survival in all patients. Table S2: Univariate and multivariate analyses of baseline factors associated with overall survival in cutaneous patients. Table S3: Propensity score matching covariates by treatment group before and after matching, patients aged <65 years with cutaneous melanoma. Table S4: Propensity score matching covariates by treatment group before and after matching, patients aged <65 years with cutaneous melanoma.
