# Supplementary material for: Effectiveness of Immune Checkpoint Inhibitor with Anti-PD-1 Monotherapy or in Combination with Ipilimumab in Younger versus Older Adults with Advanced Melanoma
Source: Curr Oncol. 2023 Sep 30;30(10):8936–47. doi: 10.3390/curroncol30100646 (PMC10605250; doi:10.3390/curroncol30100646)
Supplement: Supplementary file 1 [file curroncol-30-00646-s001.zip › curroncol-2597513-supplementary.pdf]

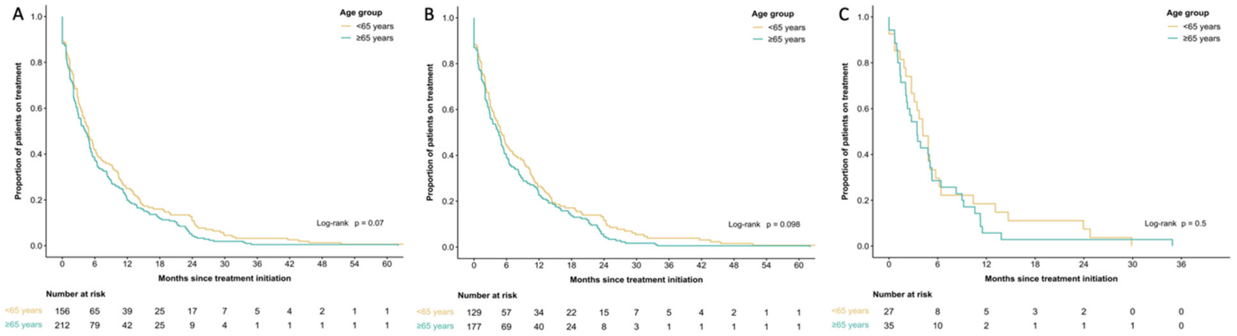

Figure S1. Kaplan-Meier curves of treatment duration amongst patients aged <65 and ≥65 years-old excluding combination immunotherapy for all melanoma (A), cutaneous melanoma only (B), and non-cutaneous melanoma only (C).

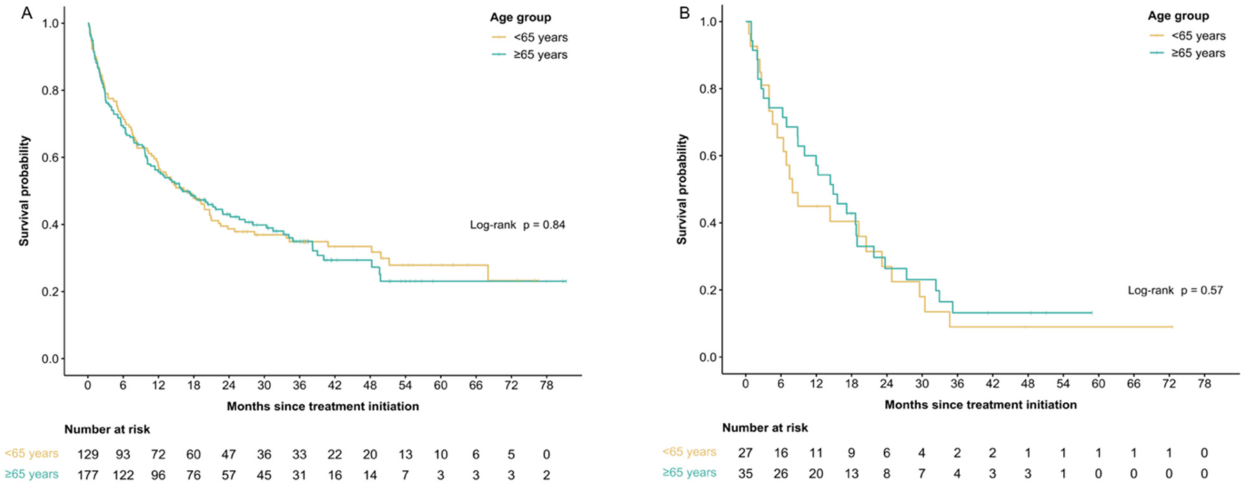

Figure S2. Kaplan – Meier curves for median overall survival amongst patients aged <65 and ≥65 years-old, when patients receiving combination immunotherapy were excluded for cutaneous melanoma patients only (A), and non-cutaneous melanoma only (B).

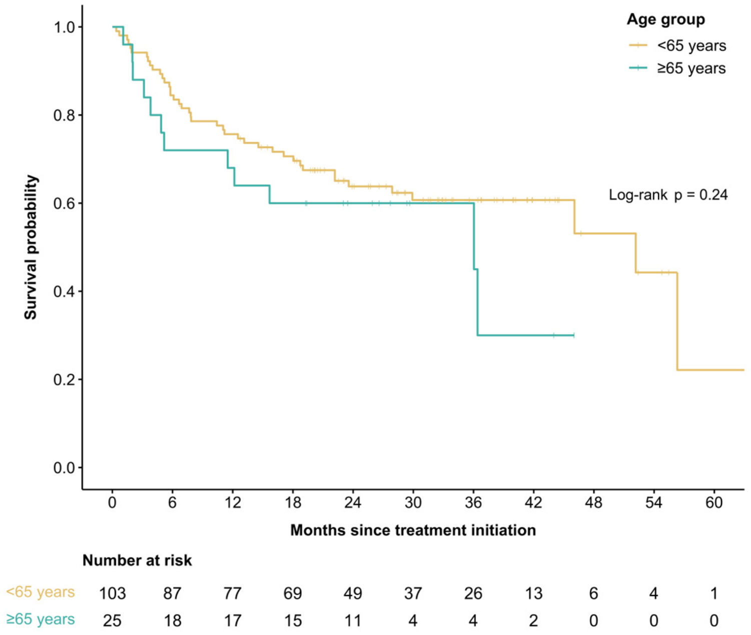

Figure S3. Kaplan – Meier curves for median overall survival amongst patients aged <65 and ≥65 years-old when stratified by patients treated with combination immunotherapy (ipilimumab and nivolumab).

Supplemental Table S1. Univariate and multivariate analyses of baseline factors associated with overall survival in all patients.

Characteristic	Univariate Cox regression model, all patients		Multivariate Cox regression model, all patients	
	OS HR (95% CI)	p-value	OS HR (95% CI)	p-value
**Age**				
<65 (reference) vs. ≥65	1.27 (1.01-1.58)	**0.04**	1.02 (0.77-1.34)	0.92
**Sex**				
Female (reference) vs. Male	1.10 (0.87-1.39)	0.43	1.21 (0.92-1.59)	0.17
**IO drug**				
Anti-PD-1 (reference) vs. Ipilimumab + nivolumab	0.48 (0.36-0.65)	**<0.001**	0.58 (0.40-0.85)	**0.006**
**Melanoma type**				
Cutaneous (reference) vs. Non-cutaneous	1.47 (1.11-1.95)	**0.008**	1.38 (1.00-1.91)	0.05
**Treatment line**				
First (reference) vs. Other	1.84 (1.46-2.32)	**<0.001**	1.44 (1.08-1.92)	**0.01**
**dNLR**				
≤3 (reference) vs. >3	2.13 (1.63-2.79)	**<0.001**	1.48 (1.07-2.04)	**0.02**
**LDH**				
Normal (reference) vs. Elevated	2.28 (1.77-2.94)	**<0.001**	2.03 (1.56-2.64)	**<0.001**
**ECOG status**				
<2 (reference) vs. ≥2	2.36 (1.76-3.18)	**<0.001**	1.54 (1.06-2.23)	**0.02**
**M stage**				
1a/1b (reference) vs. 1c/1d	1.90 (1.51-2.39)	**<0.001**	1.45 (1.11-1.90)	**0.007**

Supplemental Table S2. Univariate and multivariate analyses of baseline factors associated with overall survival in cutaneous patients.

Characteristic	Univariate Cox regression model		Multivariate Cox regression model	
	OS HR (95% CI)	p-value	OS HR (95% CI)	p-value
**Age**				
<65 (reference) vs. ≥65	1.30 (1.01-1.67)	**0.04**	1.09 (0.79-1.51)	0.59
**Sex**				
Female (reference) vs. Male	1.12 (0.85-1.46)	0.42	1.16 (0.85-1.59)	0.35
**dNLR**				
≤3 (reference) vs. >3	2.14 (1.59-2.87)	**<0.001**	1.52 (1.06-2.16)	**0.02**
**LDH**				
Normal (reference) vs. Elevated	2.32 (1.75-3.08)	**<0.001**	2.07 (1.54-2.77)	**<0.001**
**ECOG status**				
<2 (reference) vs. ≥2	2.69 (1.93-3.75)	**<0.001**	1.93 (1.28-2.92)	**0.002**
**M stage**				
1a/1b (reference) vs. 1c/1d	1.86 (1.44-2.39)	**<0.001**	1.35 (1.00-1.83)	**0.0499**
**BRAF mutation**				
No (reference) vs. Yes	1.16 (0.89-1.52)	0.27		
**IO drug**				
Anti-PD-1 (reference) vs. Ipilimumab + nivolumab	0.50 (0.36-0.70)	**<0.001**	0.67 (0.44-1.03)	0.07
**Treatment line**				
First (reference) vs. Other	1.97 (1.53-2.54)	**<0.001**	1.66 (1.20-2.29)	**0.002**

Supplemental Table S3. Propensity score matching covariates by treatment group before and after matching, patients aged <65 years with cutaneous melanoma.

Characteristic	Pre-match		SMD	Post-match		SMD
	**Anti-PD-1,** N = 102	**Ipilimumab + nivolumab,** N = 75		**Anti-PD-1,** N = 43	**Ipilimumab + nivolumab,** N = 43	
**Sex**			0.243			0.248
Female	46 (45.1%)	25 (33.3%)		17 (39.5%)	12 (27.9%)	
Male	56 (54.9%)	50 (66.7%)		26 (60.5%)	31 (72.1%)	
**ECOG status**			0.347			<0.001
<2	88 (86.3%)	72 (96.0%)		40 (93.0%)	40 (93.0%)	
≥2	14 (13.7%)	3 (4.0%)		3 (7.0%)	3 (7.0%)	
**LDH**			0.095			0.110
Normal	69 (67.6%)	54 (72.0%)		34 (79.1%)	32 (74.4%)	
Elevated	33 (32.4%)	21 (28.0%)		9 (20.9%)	11 (25.6%)	
**dNLR**			0.389			0.076
≤3	80 (78.4%)	69 (92.0%)		38 (88.4%)	39 (90.7%)	
>3	22 (21.6%)	6 (8.0%)		5 (11.6%)	4 (9.3%)	
**M stage**			0.033			0.286
1a/1b	50 (49.0%)	38 (50.7%)		22 (51.2%)	28 (65.1%)	
1c/1d	52 (51.0%)	37 (49.3%)		21 (48.8%)	15 (34.9%)	
**Treatment line**			1.279			<0.001
First	42 (41.2%)	69 (92.0%)		37 (86.0%)	37 (86.0%)	
Other	60 (58.8%)	6 (8.0%)		6 (14.0%)	6 (14.0%)	

Supplemental Table S4. Propensity score matching covariates by treatment group before and after matching, patients aged <65 years with cutaneous melanoma.

Characteristic	Pre-match		SMD	Post-match		SMD
	**Anti-PD-1,** N = 138	**Ipilimumab + nivolumab,** N = 18		**Anti-PD-1,** N = 17	**Ipilimumab + nivolumab,** N = 17	
**Sex**			0.264			<0.001
Female	38 (27.5%)	3 (16.7%)		3 (17.6%)	3 (17.6%)	
Male	100 (72.5%)	15 (83.3%)		14 (82.4%)	14 (82.4%)	
**ECOG status**			0.582			<0.001
<2	118 (85.5%)	18 (100.0%)		17 (100.0%)	17 (100.0%)	
≥2	20 (14.5%)	0 (0.0%)		0 (0.0%)	0 (0.0%)	
**LDH**			0.015			<0.001
Normal	91 (65.9%)	12 (66.7%)		12 (70.6%)	12 (70.6%)	
Elevated	47 (34.1%)	6 (33.3%)		5 (29.4%)	5 (29.4%)	
**dNLR**			0.215			<0.001
≤3	103 (74.6%)	15 (83.3%)		14 (82.4%)	14 (82.4%)	
>3	35 (25.4%)	3 (16.7%)		3 (17.6%)	3 (17.6%)	
**M stage**			0.343			<0.001
1a/1b	69 (50.0%)	6 (33.3%)		6 (35.3%)	6 (35.3%)	
1c/1d	69 (50.0%)	12 (66.7%)		11 (64.7%)	11 (64.7%)	
**Treatment line**			0.856			<0.001
First	101 (73.2%)	18 (100.0%)		17 (100.0%)	17 (100.0%)	
Other	37 (26.8%)	0 (0.0%)		0 (0.0%)	0 (0.0%)	
